# Targeted Prediction and Comprehensive Study of Stirred-Type Yogurt with Mayang *Citrus* Peel Powder Fortification Utilizing Machine Learning Approaches

**DOI:** 10.3390/foods15081427

**Published:** 2026-04-20

**Authors:** Zekui Ou, Ting Zhang, Jiali Ye, Hanyu Zhu

**Affiliations:** 1 College of Life Science, Hengyang Normal University, Hengyang 421000, China; ouzk@vip.163.com; 2 Research Institute of Farm Products Processing, Xinjiang Academy of Agricultural Science, Urumqi 830091, China; 3 Research Center of Agricultural Products Processing Engineering, Xinjiang Academy of Agricultural Science, Urumqi 830091, China; 4 Research Institute of Fruit and Vegetable, Xinjiang Academy of Agricultural Science, Urumqi 830091, China; jialiye2024@163.com

**Keywords:** machine learning, Mayang *Citrus* peel powder, stirred yogurt, physicochemical properties

## Abstract

This work examined the impact of Mayang *Citrus* peel powder (MCPP) concentrations on the physicochemical, textural, antioxidant, and flavor volatile properties of stirred yogurt while involving the application of machine learning approaches for its targeted prediction and comprehensive study. The addition of MCPP led to a dose-dependent decrease in pH, lightness, red–green color values, and water holding capacity, while increasing titratable acidity, syneresis, yellow–blue color values, viable LAB cells, polyphenol bioaccessibility, and in vitro antioxidant activity. The ratio of MCPP at 0.1% significantly increased viscosity, indicating yogurt with modified flow properties. Texture analysis revealed that yogurts fortified with 0.1% and 0.5% MCPP showed similar characteristics to the control, while a 1% concentration enhanced yogurt stability. Especially, MCPP supplementation enhanced the concentration of flavor volatiles in yogurt, and the 1% MCPP-enriched sample exhibited the highest overall quality in sensory evaluation among all formulations. A total of six machine learning predictive models were employed to comprehensively reveal the effects of MCPP addition on yogurt physicochemical and antioxidant properties, and the Lasso model achieved the highest composite score with high accuracy (R^2^ = 0.9265, RMSE = 0.0011, MSE = 1.395 × 10^−6^).

## 1. Introduction

Yogurt is popular among consumers due to its distinct taste, high nutritional value, and easy digestibility [1]. It delivers vital nutrients like protein, fat, calcium, potassium, magnesium, and vitamins, while offering health advantages such as aiding weight management, improving gastrointestinal function, strengthening the immune system, and lowering serum cholesterol levels [2,3,4]. According to Global Growth Insights, the global yogurt market is projected to reach a valuation of USD 73.72 billion by 2025, with 65% of consumers incorporating yogurt into their regular diets. Plain yogurt is traditionally considered a healthy functional food, but it contains limited amounts of bioactive compounds such as phenols and flavonoids [5,6]. Nowadays, food fortification is encouraged by the growing consumer interest to enhance the health benefits of foods since it is an efficient and cost-effective intervention [7] while yogurt is a perfect medium for such a fortification. Natural active substances are incorporated into yogurt to enhance its nutritional values and health benefits, while also altering sensory attributes (flavor, taste, color, aroma) and physical characteristics (texture, viscosity, syneresis) [4,8].

The global fruit and vegetable processing market is expected to exceed USD 598 billion by 2030, leading to a significant increase in fruit by-products [9]. Fruit by-products are rich in essential nutrients and bioactive compounds, offering considerable potential as functional ingredients in food formulations [10]. The use of fruit by-products to enhance yogurt functionality has been on the rise, and the majority are fruit pomaces and peels, including those from mulberry, apricot, peach, apple, grape, pomegranate, mango, and grapefruit [11,12,13,14,15]. These studies highlighted the potential of incorporating fruit by-products to enhance the health benefits of yogurt beyond its basic nutritional value.

Citrus fruit ranks among the world’s most widely cultivated and popular fruit crops [16,17]. China leads global citrus production, yielding 45 million tons annually [18]. Global citrus fruit production results in significant peel by-products during processing, comprising almost 50% of the wet fruit mass [19]. Nevertheless, this by-product is frequently disposed of, exacerbating environmental strain [20]. Research indicates that citrus peel is rich in health-promoting compounds, including essential oils, flavonoids, coumarins, limonoids, and alkaloids, which exhibit various biological activities such as antioxidant, anticancer, anti-inflammatory, antibacterial, hepatoprotective, antidiabetic, and neuroprotective effects [17,21]. The increasing popularity of citrus and the bioactive compounds in its peel highlight the urgent need for sustainable resource utilization of citrus peels in the industry.

Mayang *Citrus sinensis*, known as Bingtangcheng, is a superior variety developed in Mayang Miao Autonomous County, Huaihua City, Hunan Province [17,18]. The Mayang *Citrus sinensis* peel (MCP), with antioxidant and antibacterial abilities, contains a high level of carotenoid at the concentration of 227 µg/g dry weight (DW), which is higher than those in the peels of Newhall navel orange, Liangping pomelo, and Yurek lemon [20]. D-Limonene, with a pleasant lemon-like odor, exhibited high relative content (94.49–98.23%) in MCP, and it has analgesic, anti-inflammatory, antioxidant, neuroprotective, and antimicrobial effects [17,22]. Therefore, MCP has great potential as a dietary supplement to maximize functionality when combined with yogurt for its rational utilization towards value-added products.

This study examined the impact of different MCP powder concentrations (0, 0.1, 0.5, 1, and 2%) on yogurt’s flavor volatiles, structure, physicochemical properties, texture, and in vitro antioxidant activities. Furthermore, the machine learning models were applied to reveal the effects of MCPP addition on yogurt quality while constructing a useful way for its quick assessment and quality prediction. It aims to develop a theoretical basis for enhancing MCPP through deep processing and comprehensive utilization.

## 2. Materials and Methods

### 2.1. Materials

The peel of Mayang *Citrus sinensis* was freeze-dried, pulverized with a plant grinder, and sifted through a 120-mesh sieve to produce MCP powder (MCPP). The starter culture, comprising *Streptococcus thermophilus* and *Lactobacillus bulgaricus* (Angel Yeast, Yichang, Hubei, China), along with market cow milk containing 3.2% milk protein (Mengniu, Inner Mongolia, China), were obtained from a local supermarket. The reagents used were of analytical grade or authentic standard chemicals.

### 2.2. Proximate Composition Analysis

The contents of ash, crude polysaccharides, crude fat, and crude proteins of the obtained MCPP were measured according to the Association of Official Analytical Chemists methods. The examination of total phenolic content (TPC) was performed by combining MCPP water extracts with Folin–Ciocalteu’s reagent (Sigma-Aldrich, Darmstadt, Germany) and allowed to stand at room temperature for 5 min. Subsequently, 2% Na_2_CO_3_ was introduced and allowed to react for 30 min, followed by measuring the absorbance at 760 nm. A calibration curve was constructed using a gallic acid solution (Sigma Aldrich, St. Louis, MO, USA) with concentrations ranging from 0 to 100 μg/mL. TPC values were represented as GAE per gram of the sample.

### 2.3. Yogurt Preparation

Yogurt was produced by homogenizing a mixture of cow milk and 8% sucrose (g/mL) for 5 min using a SN-SJR-10 Handheld Homogenizer (Sunne, Shanghai, China). The homogenate was sterilized at 85 °C for 30 min, then cooled to 42 °C for inoculation with 1 g/L of starter culture. It was fermented at 42 °C for 6 h to coagulate the proteins, followed by cooling to 10 °C. The flavored yogurt was enhanced by incorporating MCPP at concentrations of 0.1%, 0.5%, 1%, and 2% (*w*/*v*) into the cooled plain yogurt, resulting in variants Y0.1, Y0.5, Y1, and Y2, respectively. The MCPP-fortified yogurt was gently stirred for 3 min, then distributed and stored at 4 °C in a dark environment for subsequent analysis of its physicochemical, flavor, and antioxidant properties. The plain yogurt Y0 (without the addition of MCPP) was used as the control.

### 2.4. Physicochemical and Microbiological Determinations

The pH, titratable acidity (TA), viable LAB density, and color of the yogurt samples were measured after post-ripeness for 1 d. The pH was measured using a pH meter (Ohaus, Parsippany, NJ, USA). The TA was measured using titration with NaOH and phenolphthalein as an indicator [7]. Viable LAB cell counts were assessed via serial dilution in 0.9% NaCl and streak plating on De Man-Rogosa-Sharpe agar and reported as log CFU/g [7]. Plates with 30–200 colonies were counted after 48 h of anaerobic incubation at 37 °C. Yogurt sample colors were assessed with a Konica Minolta Chroma Meter CR-400 colorimeter (Tokyo, Japan), recording the lightness (L*), red–green (a*), and yellow–blue (b*) values. The device was calibrated using a white reference standard prior to testing.

### 2.5. Structure and Rheological Properties Measurements

Syneresis and water-holding capacity (WHC) of yogurt were assessed by centrifuging 20 g samples at 5000× *g* for 5 min at 4 °C. The supernatant and sediment were weighed to express syneresis or WHC as the weight of drained whey or sediment per 100 g of yogurt [4]. The viscosity was assessed using a 150 g yogurt sample in a cup at 4 °C. A rotational viscometer (DV-II, Brookfield, Middleboro, MA, USA) equipped with a spindle no. 3 at 30 rpm was used for 1 min of rotation.

Yogurt textural properties were assessed using a TAXT Texture Analyzer (Stable Micro System, Godalming, Surrey, UK) with a backward extrusion test and a 36 mm cylindrical probe [7]. The pre-test and test speeds were both set at 1.0 mm/s, while the post-test speed was 2.0 mm/s. The trigger force was 10.0 g, and the distance measured was 10.0 mm. Texture exponent software (Exponent, version 6.11.16.0, Stable Micro Systems, Surrey, UK) was used to analyze parameters such as firmness, consistency, cohesiveness, and viscosity index.

### 2.6. Simulated In Vitro Digestion

The bioaccessibility of phenolic compounds in yogurt samples was assessed using in vitro digestion following the standardized INFOGEST protocol [23]. The simulated salivary fluid (SSF), simulated gastric fluid (SGF), and simulated intestinal fluid (SIF) were prepared and stored at 4 °C until analyses and preheated to 37 °C just before use. Stock solutions of salivary α-amylase (2505 U/mL, from human), pepsin (180 U/mL, from porcine gastric mucosa), pancreatin (800 U/mL, from porcine pancreas), and porcine bile (134 mmol/L) were freshly prepared immediately prior to the experiments [24]. All reagents and enzymes were purchased from Shanghai Macklin Biochemical Co., Ltd. (Shanghai, China).

The yogurt was mixed using a rotary shaker at 15 rpm and 37 °C with SSF (1:1; *wt*/*v*), incorporating α-amylase (75 U/mL) for 2 min at pH 7.0 to mimic oral chewing [25]. The oral bolus was combined with SGF in a 1:1 volume ratio for the gastric phase, and the pH was adjusted to 3 using 6 mmol/L HCl. Porcine pepsin (2000 U/mL) was subsequently added, which was incubated at 37 °C for 2 h under stirring at 15 rpm. The intestinal phase was started by mixing the mixture with SIF (1:1, *v*/*v*). Pancreatin and bile salts were incorporated to create a digestion mixture with final concentrations of 100 U/mL and 10 mmol/L, respectively. The sample was maintained at pH 7 and stirred at 37 °C for 2 h at 15 rpm [26]. A digestion sample, where yogurt was substituted with an equivalent weight of water, served as the blank. The MCPP water extract (2%, *wt*/*v*) was also digested. After each step, samples were collected for TPC and antioxidant capacity (AC) measurement. In the TPC assay, the yogurt sample was combined with Folin–Ciocalteu’s reagent, following the method in Section 2.2. TPC values were represented as GAE per gram of yogurt. The antioxidant activity of yogurt samples was assessed using the DPPH and ABTS radical scavenging assays, with absorbance measured at 517 nm and 734 nm, respectively, following our previous method [27]. A standard curve was constructed using an L-ascorbic acid solution (Sigma Aldrich) with concentrations ranging from 0 to 30 μg/mL. DPPH and ABTS radical scavenging activities were quantified as μg ascorbic acid equivalents (AAE) per gram of yogurt sample [24].

### 2.7. Headspace Gas Chromatography-Ion Mobility Spectrometry

Headspace gas chromatography-ion mobility spectrometry (HS-GC-IMS) with a Flavorspec^®^ instrument (G.A.S. Instrument, Berlin, Germany) was employed to analyze the volatile compounds in yogurt. A 3 g sample was sealed in a 20 mL headspace vial and incubated at 60 °C with agitation at 500 rpm for 10 min. An automated injection of a 500 μL headspace aliquot was carried out at 65 °C. Gas chromatography utilized an FS-SE-54-CB-0.5 column (0.53 mm × 15 m, 1 µm) from Restek Corporation, Bellefonte, PA, USA. Nitrogen gas with a purity exceeding 99.999% was used as the carrier gas at a flow rate of 150 mL/min. The flow rate was adjusted from an initial 2 mL/min for 2 min, increased linearly to 15 mL/min over 5 min, then to 100 mL/min over the subsequent 10 min, and finally to 150 mL/min over the next 15 min, where it was maintained for 5 min. In the IMS detector, the drift tube was maintained at 45 °C with a constant carrier gas flow rate of 150 mL/min. Volatile compounds were identified using the NIST and IMS databases, and their relative contents were quantified by normalizing peak areas [28]. Each group underwent a minimum of three measurements.

### 2.8. Sensory Evaluation

Twenty trained panelists (ISO Standard 22935-3 [29]) evaluated the sensory attributes of the yogurts. The panel consisted of 20 individuals (10 women and 10 men) aged 18 to 40, comprising staff and graduate and undergraduate students from the Research Institute of Farm Product Processing at Hengyang Normal University. All evaluators willingly took part and signed a consent form, which was reviewed and approved by Hengyang Normal University (No. 2025LL24). Samples were removed from the refrigerator and immediately presented to panelists for sensory evaluation at room temperature. To prevent biased responses, each sample was coded with a random three-digit number. The ratings were based on a nine-point hedonic scale: 1 = highly dislike, 2 = dislike very much, 3 = rather dislike, 4 = dislike a little, 5 = neutral, 6 = quite like, 7 = rather like, 8 = like a lot, and 9 = like very much [30]. Yogurt sensory parameters were assessed based on color, fermented odor, texture, taste quality, and overall acceptance. The values obtained for each sensory perception were given as averages of the number of panelists’ values for each sample.

### 2.9. Construction and Assessment of Predictive Models

The data preprocessing and dimensionality reduction were performed firstly. Principal component analysis (PCA) was employed for data dimensionality reduction and feature extraction. Initially, the original feature matrix underwent Z-score standardization to achieve zero mean and unit variance for each feature, eliminating dimensional influence. The standardization formula is:

(1) $$X_{std}=\frac{X-\mu }{\sigma }$$

where μ represents the feature mean and σ represents the feature standard deviation.

Subsequently, singular value decomposition was performed on the standardized data matrix to compute eigenvalues and eigenvectors of the covariance matrix. The principal components were selected as model input features based on the criterion of cumulative variance contribution rate ≥90%. The principal component score matrix was calculated as:

(2) $$T=X_{std}\;\times W$$

where W is the loading matrix (eigenvector matrix).

Six representative machine learning regression algorithms were systematically compared: Support Vector Machine (SVM), XGBoost, Gradient Boosted Regression Trees (GBRT), Ridge Regression, Lasso Regression, and ElasticNet. Key parameter settings and detailed configuration information for six machine learning algorithms (Table S1). The bootstrap resampling method was employed for model validation. Training sets were constructed by randomly sampling n samples with replacement from the original dataset (n samples), with unselected samples (out-of-bag, OOB) serving as validation sets. This process was repeated 100 times, yielding 100 independent training-validation partitions. Means and standard deviations of performance metrics were calculated to effectively assess model stability and generalization capability. Three metrics were employed to comprehensively evaluate model performance: Coefficient of Determination (R^2^), Root Mean Square Error (RMSE), and Mean Square Error (MSE). To balance model interpretability and prediction accuracy, a weighted composite scoring system was constructed:

(3) $$Score=0.6\times R_{norm}^{2}+0.4\times {RMSE}_{norm}$$

where normalization formulas are:

(4) $$R_{norm}^{2}=\frac{R^{2}-min(R^{2})}{\max\left(R^{2}\right)-min(R^{2})}$$

(5) $${RMSE}_{norm}=\frac{\frac{1}{RMSE}-min(\frac{1}{RMSE})}{\max\left(\frac{1}{RMSE}\right)-min(\frac{1}{RMSE})}$$

All analyses were conducted in the MATLAB R2024a environment. The Statistics and Machine Learning Toolbox was utilized for SVM, Ridge regression, Lasso, and ElasticNet implementations, the TreeBagger function for XGBoost implementation, and the fitrensemble function for GBRT implementation. Visualizations employed unified color schemes to ensure consistent and interpretable presentation of results.

### 2.10. Statistical Analysis

Data means were derived from three independent samples. Results are presented as mean ± standard deviation. Statistical significance was determined by one-way analysis of variance (ANOVA) and performed using SPSS 23.0 software (IBM Corporation, New York, NY, USA). Duncan’s post hoc test was used to assess significant differences at the specified significance level. Values of *p* < 0.05 were considered statistically significant. Spearman’s correlation analysis was used to evaluate nonparametric correlations between variables, with correlation coefficients and *p*-values computed using IBM SPSS Statistics 27.0. PCA and correlation heatmaps were created using Origin 2022 (OriginLab, Northampton, MA, USA).

## 3. Results and Discussion

### 3.1. Characterization of the General Nutritional Value of MCPP

The MCPP contains 91.82 ± 0.31 g crude polysaccharides, 3.99 ± 0.28 g protein, 1.55 ± 0.13 g fat, and 2.64 ± 0.01 g ash per 100 g DW. The TPC of its aqueous extract is 10.14 ± 0.30 mg gallic acid equivalent (GAE)/g DW.

### 3.2. Effect of MCPP on Yogurt Color

**Figure 1:**
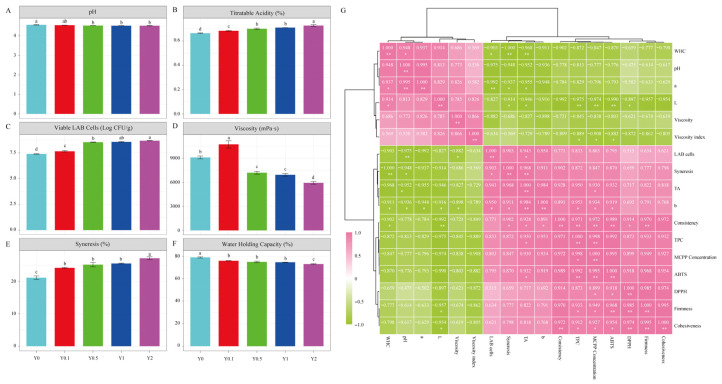
The physicochemical and microbiological parameters of stirred yogurt fortified with Mayang *Citrus sinensis* L. Osbeck peel powder (MCPP). (**A**) pH. (**B**) Titratable acidity. (**C**) Viable LAB cells. (**D**) Viscosity. (**E**) Syneresis. (**F**) Water holding capacity. (**G**) Correlation heatmaps. Y0: the 0% MCPP-fortified yogurt (as the control, the light-blue column); Y0.1: 0.1% MCPP-fortified yogurt, the red column; Y0.5: 0.5% MCPP-fortified yogurt, the green column; Y1: 1% MCPP-fortified yogurt, the dark-blue column; Y2: 2% MCPP-fortified yogurt, the purple column. Different lowercase letter superscripts indicate statistically significant differences at *p* < 0.05 between the yogurts fortified with different amounts of MCPP. The correlation between measured parameters and MCPP ratio was determined by calculating the Pearson correlation coefficients (*r*). The correlation heat map represents the *r* values, with red squares representing positive and green negative correlations. The significance of *r* values is indicated with * and ** for significant levels of 0.05 and 0.01, respectively.

Color is a crucial factor in yogurt quality, influencing consumer acceptance and palatability [4,7]. Table 1 outlines the color changes in yogurt fortified with different ratios of MCPP. The addition of varying amounts of MCPP significantly affected the color parameters of the stirred flavored yogurt samples (*p* < 0.001). The MCPP concentration was significantly negatively correlated with the L* value (*r* = −0.974, *p* < 0.01) and positively correlated with the b* value (*r* = 0.934, *p* < 0.05) (Figure 1G). The MCPP yogurt showed decreased L* and a* values and an increased b* value compared to the control group. This suggests a decrease in yogurt brightness, accompanied by increased greenness and yellowness. Yogurt color variations may be attributed to the presence of chlorophylls and carotenoids, the two primary pigments in citrus peel [31]. Compared to control samples, the overall color change (ΔE) of the MCPP-fortified yogurts was in the range of 3.78–27.16, which revealed a dose-dependent manner. Although consumers may tolerate slight hue or saturation variations within expected color ranges [32], a high MCPP concentration in yogurts significantly altered the color.

### 3.3. Effect of MCPP on pH, TA, Viable LAB Cells, Viscosity, Syneresis, and WHC

Yogurt pH and TA indicate organic acid levels, assessing microbial growth and the impact of organic acids on flavor [33]. The recommended pH range for yogurt is pH 4.6 or lower [34]. Herein, the yogurt samples exhibited a pH range of 4.52 to 4.56 and a TA range of 0.66% to 0.73% (Figure 1A,B). Yogurt pH values in different treatments were lower than the control (Y0), with a corresponding increase in TA (*r* = 0.930, *p* < 0.05). The starter culture produced more lactic acid, which might be due to the nutrients in MCPP [34]. Lactic acid plays a crucial role in yogurt formation by coagulating milk protein and is primarily responsible for its characteristic acidic taste and aroma, enhancing its flavor [35]. Microbiological evaluation confirmed that the variations in pH and TA were associated with the growth of the yogurt starter (Figure 1C). The LAB count results further confirm that MCPP enhanced the growth of LAB.

FAO/WHO guidelines state that fermented beverages should contain more than 7 log CFU/g of LAB, and the high viable probiotic counts in yogurts can effectively meet the requirements for probiotic action in the host [27]. It seems that incorporation of MCPP in yogurt is an effective strategy to enhance probiotic viability. The LAB counts revealed a promotion effect of MCPP, and the highest cell density occurred in the 2–MCPP fortified yogurt as predicted. The lactic acid content decreases the pH level, creating a more acidic yogurt medium [8]. Polysaccharides and polyphenols added to yogurt act as prebiotics, enhancing lactic and organic acid production by LAB, while also protecting probiotics in simulated gastric and bile environments, thereby benefiting the host [27,36]. Likewise, yogurts fortified with mango and banana peel powder supported LAB cell viability during storage [15].

Yogurt’s viscosity influences its mouthfeel [37], and low viscosity is a key defect affecting consumer acceptance of yogurt products [27]. With increasing concentration of MCPP, values of viscosity significantly increased initially in Y0.1 and then decreased (*p* < 0.05, Figure 1D). The initial rise at a 0.1% MCPP ratio can be attributed to phenolic compounds interacting with proteins, promoting protein gel network formation and resulting in a thicker, more viscous yogurt medium [8]. The levels of viscosity were closely related to the gel strength of yogurt [6]. Both the increasing MCPP and the decreasing pH contributed to the weakness of the gel network [7], which resulted in the lower viscosity.

Yogurt’s shelf life and consumer acceptability are significantly influenced by WHC and syneresis [4]. The addition of MCPP at different concentrations affected the syneresis and WHC of yogurt (Figure 1E,F). The syneresis of yogurt containing MCPP significantly increased (*p* < 0.001) with the addition of MCPP, while the levels of WHC changed vice versa. Syneresis is a frequent defect in yogurt production that adversely impacts consumer perception [27]. Increasing the ratio of MCPP led to higher syneresis and lower WHC. Similar results were observed in yogurts fortified with orange peel, grape, mulberry, and carob molasses, likely due to increased acidity impacting both soluble protein complexes and micelle-bound structures, thereby reducing the yogurt gel’s capacity to retain whey [34]. Additionally, it showed that the supplemented powder could disrupt protein-water bonds, leading to liquid expulsion. To sum up, adding MCPP into yogurt seems to have negative effects on WHC and viscosity [37].

### 3.4. Effect of MCPP on Yogurt Texture Profile

Texture is crucial for both the structural integrity and sensory appeal of food products to consumers [38]. In the food industry, yogurt’s textural and rheological properties are essential for its stability and consumer satisfaction [2]. Firmness refers to a product’s moderate resistance to deformation, cohesiveness indicates its tendency to stick together, and consistency pertains to the firmness, thickness, or viscosity of a liquid or semi-solid [32,39]. Texture profile analysis revealed that MCPP-fortified yogurt demonstrated significantly greater firmness, cohesiveness, and consistency than the control (*p* < 0.001, Table 2). The viscosity index showed similar changing trends with the viscosity parameter. Incorporating MCPP enhanced the firmness, consistency, and cohesiveness of yogurt in a dose-dependent manner, except for a slight, non-significant decrease in cohesiveness at Y0.5. The results of the correlation analysis (Figure 1G) further confirmed their close relationships, where MCPP concentration had notable positive relationships with firmness (*r* = 0.949, *p* < 0.05), cohesiveness (*r* = 0.927, *p* < 0.05), and consistency (*r* = 0.972, *p* < 0.01), and was inversely related to the viscosity index (*r* = −0.908, *p* < 0.05).

Firmness is a key indicator of yogurt texture, reflecting the stability of its gel network [4]. The increased firmness likely resulted from higher syneresis, reducing the yogurt’s water content and subsequently decreasing its softness [27]. The increased protein and dry matter content with MCPP addition might also account for the observed differences, aligning with findings from set-type yogurt enriched with pineapple pomace powder [40].

The consistency value indicates the product’s density. Measurement results suggest that a high consistency value signifies a dense product, enhancing consumer acceptance [38,41]. Yogurt samples with MCPP exhibited significantly greater consistency compared to the control (*p* < 0.05). The viscosity index results corresponded with the viscosity data, showing an initial increase in Y0.1 followed by a decrease. A lower viscosity index suggests reduced flow resistance, indicating increased fluidity under specific conditions [38]. In summary, the 0.1% and 0.5% MCPP-fortified yogurts exhibited similar texture profiles compared with the control, and a 1% concentration may positively influence yogurt stability and consumer acceptance since it displayed a similar viscosity index and higher values of firmness, consistency, and cohesiveness.

### 3.5. Effect of MCPP on Yogurt TPC and AC Bioaccessibility

Table 3 demonstrates a notable, dose-dependent increase in the phenolic content and antioxidant capacity of yogurt upon the addition of MCPP (*p* < 0.05). Elevating MCPP concentration notably increased yogurt’s TPC and its DPPH and ABTS scavenging activities (*p* < 0.05), as corroborated by Pearson correlation analysis (*r* = 0.998 for TPC, *r* = 0.899 for DPPH, and *r* = 0.995 for ABTS, *p* < 0.05) (Figure 1G). The elevated TPC and AC validated the fortification process in the fortified yogurt. Yogurt enriched with the highest percentage of MCPP demonstrated the greatest TPC and antioxidant activity, indicating potential for enhancing yogurt’s biological activity through citrus by-product enrichment.

In our bodies, antioxidants reduce the damage induced by excessive accumulation of reactive oxygen species, which cause several chronic diseases, including cardiovascular and neurological diseases, obesity, diabetes, and cancer [6,33]. The World Health Organization’s 2023 report estimates that chronic diseases cause 41 million deaths each year, representing about 70% of global mortality. Dietary antioxidants are widely recognized as crucial for preventing and managing chronic diseases [6]. Our findings indicate that fortifying yogurt with MCPP is a safe and effective method to create antioxidant-rich functional yogurt, meeting consumer demands and enhancing yogurt’s health benefits. MCPP serves as a primary antioxidant source, with its water extract exhibiting a TPC value of 10.14 ± 0.30 mg gallic acid equivalent (GAE)/g DW. This value surpasses those reported for pineapple peel [42], mulberry pomace [13], and mushroom fruiting body [7].

The health impacts of phenolics are dependent on their bioavailability, as they must be ingested, absorbed in the intestines, and digested within the body to be effective [43]. Phenolic compounds need to be released from plant cell vacuoles into the gastrointestinal tract to exhibit their biological activity [44]. Polyphenols, as potent antioxidants, can help maintain redox balance in the gastrointestinal tract without being absorbed [24]. Thus, measurements of TPC and AC of yogurt were conducted after each step of in vitro digestion.

After oral digestion, the DPPH scavenging activity in yogurt samples showed a slight increase (*p* > 0.05, Table 3), while the TPC and ABTS scavenging activity remained statistically unchanged compared to the undigested samples. However, the values were significantly decreased in all yogurts after gastric and intestinal digestion (*p* < 0.05). The consistent trends of TPC and AC suggest that phenolics undergo degradation or transformation during in vitro digestion. The reduced retention rates may be attributed to phenolics and proteins, or other macromolecules, forming high molecular weight complexes through covalent or non-covalent bonds [43]. These complexes precipitate and are removed during centrifugation after intestinal digestion. In addition, polymerization and glycosylation of phenolics can decrease their bioavailability [45]. The bioavailability of phenolics can vary due to many factors [43]. In vitro digestion of dairy products supplemented with spinach exhibited similar decreasing trends in TPC and AC [46], while coffee-fortified yogurt showed increasing patterns [47].

It is worth mentioning that though yogurt itself has certain antioxidant properties [48], the TPC and AC of 2% MCPP water extract (M2) significantly decreased compared with those in Y1 and Y2 during all digestion phases. The yogurt matrix effectively delivers phenolic compounds and improves their retention during digestion. Additional research is required to determine the functional properties of MCPP yogurt through both in vitro and in vivo trials.

### 3.6. Effect of MCPP on Yogurt Flavor Volatiles

HS-GC-IMS, a novel analytical method distinguishing ionized compounds by their migration rates in an electric field, offers exceptional sensitivity, operational simplicity, high separation capacity, selectivity, and flavor substance visualization, which has been utilized for volatile analysis in various juices [49]. HS-GC-IMS is mainly used for the qualitative and relative quantitative analysis of volatile compounds, which is sufficient for evaluating their influence on overall aroma [28]. To clarify the notable alterations in aroma profiles of fortified yogurt, HS-GC-IMS analysis was conducted immediately.

**Figure 2:**
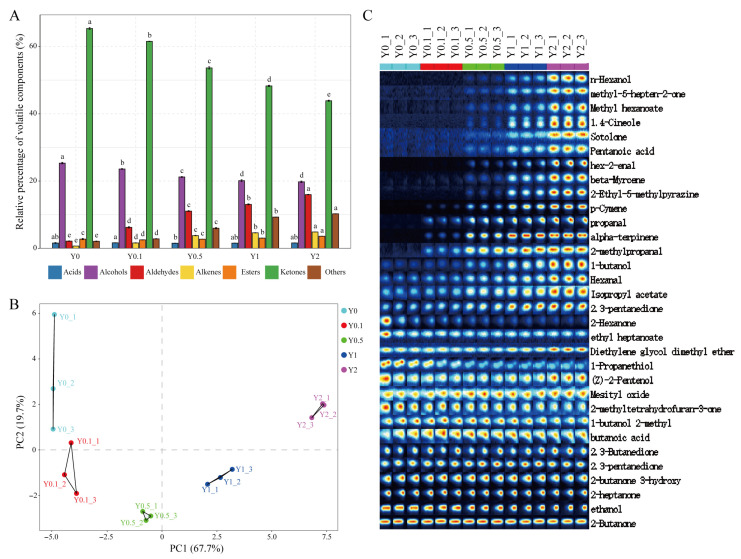
Volatile components analysis of stirred yogurt fortified with Mayang *Citrus sinensis* L. Osbeck peel powder (MCPP) based on HS-GC-IMS. (**A**) Changes in significant aroma fractions. (**B**) Principal component analysis. (**C**) Fingerprint spectra; each row represents one sample. Values are presented as mean ± standard deviation (n = 3). Y0.1: 0.1% MCPP-fortified yogurt; Y0.5: 0.5% MCPP-fortified yogurt; Y1: 1% MCPP-fortified yogurt; Y2: 2% MCPP-fortified yogurt. Different lowercase letter superscripts indicate statistically significant differences at *p* < 0.05 between the yogurts fortified with different amounts of MCPP.

Table S2 identifies 32 flavor volatiles in yogurt, including four aldehydes, ten ketones, six alcohols, two acids, four esters, and two alkenes. As depicted in Figure 2A, ketones emerged as the most relatively abundant volatile components and were followed by aldehydes and alcohols. The relative contents of ketones and alcohols showed decreasing trends along with the addition of MCPP (*p* < 0.05), while aldehydes, alkenes, and esters increased in dose-dependent manners (*p* < 0.05). Ketones, produced by oxidizing free fatty acids and degrading amino acids, directly impact the aroma of fermented dairy products [50]. Aldehydes are derived from amino acids via transamination or Strecker degradation [51], while esters, formed through the esterification of alcohols and organic acids, are key flavoring agents known for their fruity or floral aromas [52].

The relative peak intensities of hexanal, 2-methylpropanal, propanal, hex-2-enal, 2-methyltetrahydrofuran-3-one, methyl-5-hepten-2-one, 1-butanol, n-hexanol, pentanoic acid, isopropyl acetate, methyl hexanoate, alpha-terpinene, beta-myrcene, p-cymene, and 2-ethyl-5-methylpyrazine increased proportionally with the MCPP ratio (*p* < 0.05), whereas 2-hexanone decreased as MCPP was added (*p* < 0.05, Figure 2C). Among them are propanal, 1-butanol, pentanoic acid, and 2-hexanone (floral, fruity), which have been identified in plain yogurt [53]. The increased volatile compounds might contribute to the improvement of yogurt flavor since most of them provided fresh, fruity, and nutty aromas, e.g., hexanal (grassy, green, soapy), 2-methylpropanal (malty), hex-2-enal (green, fresh, fruity), hexanol (green, cut grass), methyl hexanoate (fruity, pineapple, sweaty, cheesy), terpinene (citrusy, woody, lemon-like), p-cymene (aromatic), 2-methyltetrahydrofuran-3-one (nutty, astringent, creamy, almond), and 2-ethyl-5-methylpyrazine (nutty) [54,55,56,57,58]. It is well known that 2-butanone, 3-hydroxy and 2,3-butandione are important and dominant compounds in providing a creamy, sweet, and butter-like flavor in yogurt [51], and these compounds were found to have no obvious difference in intensity among all samples.

Principal Component Analysis (PCA) was conducted using the signal intensities of volatile compounds identified through HS-GC-IMS to assess the notable compositional differences between the plain yogurt and MCPP-enriched groups (Figure 2B). The findings indicated that all samples were within the 95% confidence interval. PC1 and PC2 accounted for 67.7% and 19.7% of the variance, respectively. This effectively highlighted the key characteristics that differentiate the various yogurt sample groups. The addition of MCPP significantly altered the volatile flavor compound composition in yogurts, creating a clear distinction between the control and fortified groups.

### 3.7. Effect of MCPP on Yogurt Sensory Properties

**Figure 3:**
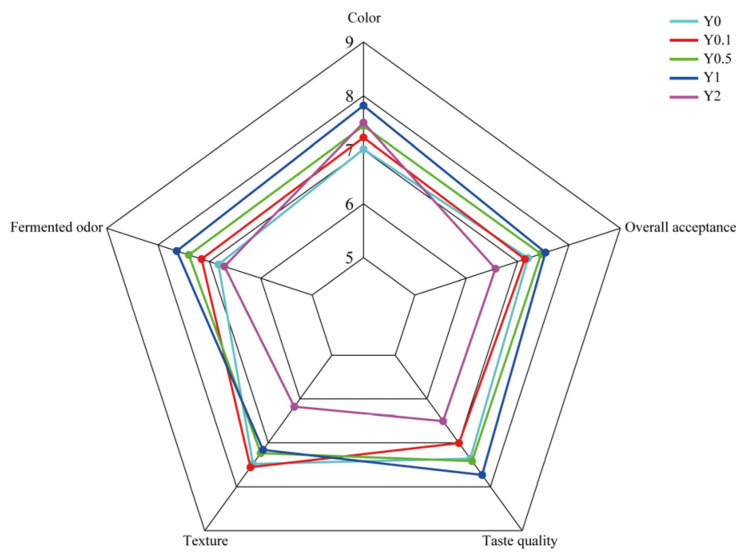
Sensory analysis of stirred yogurt fortified with Mayang *Citrus sinensis* L. Osbeck peel powder (MCPP). Y0: the 0% MCPP-fortified yogurt (as the control); Y0.1: 0.1% MCPP-fortified yogurt; Y0.5: 0.5% MCPP-fortified yogurt; Y1: 1% MCPP-fortified yogurt; Y2: 2% MCPP-fortified yogurt.

Sensory evaluation is a key determinant of consumer food acceptance [59]. The descriptive sensory analysis attributes of yogurt were described by five attributes, and results were plotted as a radar chart (Figure 3). The inclusion of MCPP enhanced the yogurt’s color, as fortified samples received significantly higher color ratings than the control (*p* < 0.05). The incorporation of MCPP at 0.5% and 1% notably enhanced the fermented odor and taste quality of yogurt (*p* < 0.05), likely due to the flavor compounds present in MCPP. The 1% MCPP-fortified yogurt achieved the highest overall acceptance, with a statistically significant difference (*p* < 0.05), indicating its potential suitability for industrial production. These findings indicate that incorporating MCPP into yogurt at concentrations of 0.5% to 1% results in products with optimal ratings across all evaluated characteristics.

A 2% concentration of MCPP supplementation adversely affected the fermented odor, texture, taste quality, and overall acceptance, resulting in low consumer approval. The ratio higher than 1% appeared to have a negative impact on the sensory properties of yogurt. It could be the result of the syneresis and texture properties of Y2. Also, Y2 gained the lowest taste score, which might be because of higher TPC. Yogurts containing 0.4% black garlic polyphenols received the lowest sensory scores for flavor, texture, taste, and color, whereas no significant differences were observed in these parameters for yogurts with 0.1–0.3% black garlic polyphenols [60].

### 3.8. Machine Learning Models to Predict the Effect of MCPP Ratio on the Physicochemical and Antioxidant Properties of Yogurt

Machine learning, a subset of artificial intelligence, plays important roles in improving food processes, predicting food quality, and accelerating new food product development, which benefits the transformation of the food industry [61]. By integrating modern flavor analysis methods with machine learning in meat, fruit, and fermented food, the process becomes more time-saving and objective compared to manual analysis, allowing for accurate predictions of the flavors of unknown food samples [62].

In this study, the corresponding variance explained rates of principal component analysis were 79.9%, 9.7%, and 5.2%, respectively (Figure S1, Table S3). The cumulative variance contribution rate exceeded the preset 90% threshold. Therefore, the first three principal components were selected as input features for subsequent machine learning models, effectively reducing dimensionality while preserving most information from the original dataset.

**Figure 4:**
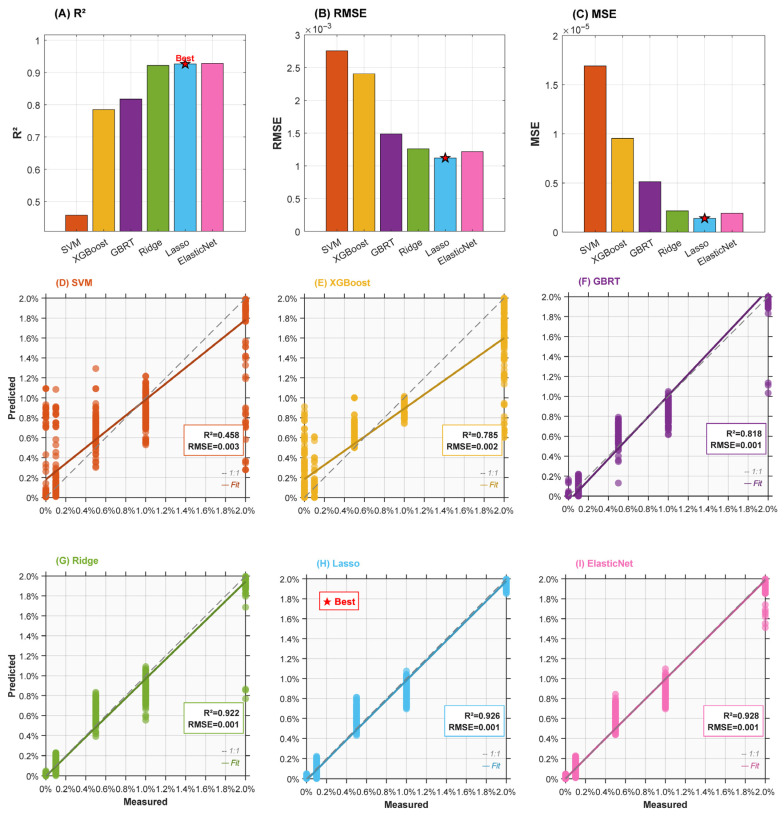
Performance evaluation comparison of six machine learning models and measured vs. predicted value fitting plots for six models. (**A**) Comparison of coefficient of determination (R^2^): higher values indicate stronger explanatory power of the model for the target variable; (**B**) Comparison of root mean square error (RMSE): lower values indicate higher prediction accuracy; (**C**) Comparison of mean square error (MSE): lower values indicate smaller prediction deviation. A red pentagram marks the model with the optimal composite score. (**D**–**I**) presents the aggregated prediction results from 100 bootstrap validations for SVM, XGBoost, GBRT, Ridge, Lasso, and ElasticNet models, respectively. The *x*-axis represents measured values, the *y*-axis represents predicted values, the grey dashed line indicates the ideal y = x prediction line, and the solid-colored lines represent the actual fitted regression lines for each model. Mean R^2^ and RMSE values are annotated in the upper right corner of each subpanel. ★ marks the model with the optimal composite score. Models follow a unified color scheme: SVM (orange-red), XGBoost (golden), GBRT (purple), Ridge (green), Lasso (sky blue), and ElasticNet (pink).

Mean performance metrics of six machine learning models across 100 bootstrap validations are presented in Table S4. The R^2^ assesses the linear association between the model’s predictions and the true values, whereas the RMSE reflects the standard deviation of the differences between these predicted and actual results [32]. Composite scores (60% R^2^ + 40% normalized RMSE) revealed the following model performance ranking: Lasso > ElasticNet > Ridge > GBRT > XGBoost > SVM. Lasso achieved the highest composite score of 0.9975, with an R^2^ of 0.9265, an RMSE of 0.0011, and an MSE of 1.395 × 10^−6^. Both metrics significantly outperformed other comparative models (*p* < 0.001). This model was identified as the optimal prediction model in this study. Measured-predicted scatter plots for six models (Figure 4) exhibited all models demonstrating significant positive correlations between predicted and measured values (*r* > 0.85, *p* < 0.001), with scatter points predominantly distributed along the y = x diagonal line, indicating successful capture of major variation trends in the target variable. Hence, it can be inferred that the developed Lasso model is appropriate for predicting the physicochemical and antioxidant characteristics of MCPP yogurt.

## 4. Conclusions

The MCPP-enriched yogurt had decreased pH, lightness, red–green color values, and WHC, and increased titratable acidity, syneresis, yellow–blue color values, viable LAB cells, polyphenol bioaccessibility, and in vitro antioxidant activity. The addition of 0.1% MCPP significantly increased viscosity but declined at the higher addition. Texture profiles indicated comparable trends for the control, 0.1%, and 0.5% MCPP-fortified yogurts, whereas the 1% concentration enhanced yogurt stability. Total phenol content increased from 0.87 to 2.36 mg GAE/100 g, and antioxidant capacity rose from 0.65 to 2.34 mg AAE/100 g after MCPP supplement. HS-GC-IMS analysis revealed that the volatile flavor profile differed from plain yogurt, indicating flavor modification. Except for Y2, sensory evaluation revealed significant increases in fermented odor and overall acceptance, and the 1% MCPP-enriched sample displayed the most favorable overall quality. Finally, six machine learning predictive models were employed to comprehensively reveal the effects of MCPP addition on yogurt physicochemical and antioxidant properties, and Lasso achieved the highest composite score with high accuracy (R^2^ = 0.9265, RMSE = 0.0011, MSE = 1.395 × 10^−6^). These findings suggest the potential application of machine learning approaches in targeted prediction and comprehensive study of yogurt products and MCPP in serving as a functional ingredient. Further research is necessary to determine the composition and content of bioactive compounds in MCPP that contribute to health benefits, explore the microbial change during the storage of MCPP-fortified yogurt for edible security, optimize the machine learning methods through the improvement of algorithms or the use of hybrid models to improve the predictability, and eventually provide more innovative and appealing yogurt products to align with the demands of consumers and dairy product manufacturers.

## Figures and Tables

**Table 1:** Color analysis of the stirred yogurt incorporated with Mayang *Citrus sinensis* L. Osbeck peel powder (MCPP).

Treatment	Sample	L*	a*	b*	ΔE
Y0	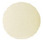	92.52 ± 0.36 ^a^	−1.76 ± −0.02 ^a^	10.18 ± 0.27 ^e^	-
Y0.1	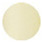	91.64 ± 0.24 ^b^	−2.72 ± −0.04 ^b^	13.71 ± 0.21 ^d^	3.78 ± 0.36 ^d^
Y0.5	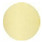	90.85 ± 0.59 ^c^	−4.25 ± −0.04 ^c^	24.48 ± 0.72 ^c^	14.62 ± 0.94 ^c^
Y1	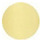	90.63 ± 0.03 ^c^	−4.48 ± −0.09 ^d^	31.86 ± 0.24 ^b^	21.93 ± 0.53 ^b^
Y2	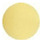	88.32 ± 0.49 ^d^	−4.63 ± −0.08 ^e^	36.86 ± 0.45 ^a^	27.16 ± 0.15 ^a^

All values are means ± SD (n = 3). Different lowercase letter superscripts in the same line indicate statistically significant differences at *p* < 0.05 between the yogurts fortified with different amounts of MCPP (One-way Analysis of Variance followed by Duncan post hoc test). L* = lightness; a* = red (+)-green (−) color; b* = yellow (+)-blue (−) color. Y0: the 0% MCPP-fortified yogurt (as the control); Y0.1: 0.1% MCPP-fortified yogurt; Y0.5: 0.5% MCPP-fortified yogurt; Y1: 1% MCPP-fortified yogurt; Y2: 2% MCPP-fortified yogurt.

**Table 2:** Texture analysis of the stirred yogurt incorporated with Mayang *Citrus sinensis* L. Osbeck peel powder (MCPP).

Treatment	Firmness (g)	Consistency (g × s)	Cohesiveness (g)	Viscosity Index (g × s)
Y0	59.86 ± 1.77 ^c^	325.75 ± 19.07 ^c^	14.96 ± 0.85 ^c^	60.07 ± 4.84 ^b^
Y0.1	63.92 ± 1.93 ^bc^	361.80 ± 15.89 ^bc^	18.42 ± 1.00 ^b^	72.62 ± 7.65 ^a^
Y0.5	64.58 ± 3.33 ^bc^	369.40 ± 29.62 ^b^	17.91 ± 0.77 ^bc^	61.60 ± 2.88 ^b^
Y1	67.67 ± 0.81 ^b^	388.99 ± 16.10 ^b^	18.75 ± 0.72 ^b^	55.69 ± 3.55 ^b^
Y2	91.93 ± 7.15 ^a^	464.62 ± 6.99 ^a^	30.35 ± 3.05 ^a^	39.39 ± 6.59 ^c^

All values are means ± SD (n = 3). Different lowercase letter superscripts in the same line indicate statistically significant differences at *p* < 0.05 between the yogurts fortified with different amounts of MCPP (One-way Analysis of Variance followed by Duncan post hoc test). Y0: the 0% MCPP-fortified yogurt (as the control); Y0.1: 0.1% MCPP-fortified yogurt; Y0.5: 0.5% MCPP-fortified yogurt; Y1: 1% MCPP-fortified yogurt; Y2: 2% MCPP-fortified yogurt.

**Table 3:** Total phenolic content and antioxidant assays of the stirred yogurt incorporated with Mayang *Citrus sinensis* L. Osbeck peel powder (MCPP) before and after in vitro digestion.

Parameters	Treatment					
	Y0	Y0.1	Y0.5	Y1	Y2	M2
TPC (mg gallic acid/g) ^1^						
Undigested	0.87 ± 0.01 ^f,A^	1.00 ± 0.06 ^e,A^	1.31 ± 0.03 ^d,A^	1.70 ± 0.03 ^c,A^	2.36 ± 0.04 ^a,A^	2.15 ± 0.06 ^b,A^
Oral phase	0.84 ± 0.04 ^e,A^	0.94 ± 0.02 ^e,A^	1.32 ± 0.07 ^d,A^	1.70 ± 0.05 ^c,A^	2.39 ± 0.12 ^a,A^	2.14 ± 0.04 ^b,A^
Gastric phase	0.61 ± 0.01 ^e,B^	0.62 ± 0.01 ^e,B^	1.24 ± 0.01 ^c,A^	1.55 ± 0.05 ^b,B^	1.69 ± 0.05 ^a,B^	1.16 ± 0.04 ^d,B^
Intestinal phase	0.43 ± 0.01 ^d,C^	0.44 ± 0.01 ^d,C^	0.93 ± 0.00 ^b,B^	0.93 ± 0.02 ^b,C^	1.00 ± 0.01 ^a,C^	0.65 ± 0.01 ^c,C^
AAE_DPPH_ (mg ascorbic acid/g) ^2^						
Undigested	0.80 ± 0.01 ^d,A^	0.81 ± 0.01 ^cd,A^	0.81 ± 0.02 ^cd,AB^	0.86 ± 0.03 ^c,A^	1.86 ± 0.06 ^a,A^	1.02 ± 0.04 ^b,A^
Oral phase	0.83 ± 0.07 ^bc,A^	0.81 ± 0.06 ^c,A^	0.92 ± 0.10 ^bc,A^	1.05 ± 0.12 ^b,B^	1.88 ± 0.23 ^a,A^	0.93 ± 0.00 ^bc,B^
Gastric phase	0.53 ± 0.01 ^c,B^	0.55 ± 0.00 ^c,B^	0.75 ± 0.01 ^b,B^	0.79 ± 0.06 ^b,B^	1.12 ± 0.06 ^a,B^	0.81 ± 0.02 ^b,C^
Intestinal phase	0.29 ± 0.07 ^b,C^	0.38 ± 0.02 ^b,C^	0.40 ± 0.08 ^b,C^	0.44 ± 0.02 ^b,C^	0.71 ± 0.16 ^a,C^	0.43 ± 0.02 ^b,D^
AAE_ABTS_ (mg ascorbic acid/g) ^3^						
Undigested	0.65 ± 0.00 ^f,AB^	0.86 ± 0.01 ^e,A^	1.13 ± 0.07 ^d,A^	1.42 ± 0.02 ^c,A^	2.34 ± 0.10 ^a,A^	1.77 ± 0.13 ^b,A^
Oral phase	0.68 ± 0.01 ^d,A^	0.84 ± 0.03 ^d,A^	1.20 ± 0.06 ^c,A^	1.45 ± 0.08 ^c,A^	2.29 ± 0.41 ^a,A^	1.79 ± 0.18 ^b,A^
Gastric phase	0.62 ± 0.03 ^d,B^	0.68 ± 0.08 ^d,B^	0.84 ± 0.01 ^c,B^	0.92 ± 0.01 ^b,B^	1.13 ± 0.02 ^a,B^	0.98 ± 0.04 ^b,B^
Intestinal phase	0.26 ± 0.05 ^d,C^	0.35 ± 0.01 ^c,C^	0.49 ± 0.04 ^b,C^	0.60 ± 0.07 ^a,C^	0.64 ± 0.04 ^a,C^	0.26 ± 0.04 ^d,C^

All values are means ± SD (n = 3). Different lowercase letter superscripts in the same line indicate statistically significant differences at *p* < 0.05 between the samples fortified with different amounts of MCPP (One-way Analysis of Variance followed by Duncan post hoc test). Different capital letter superscripts in the same column indicate statistically significant differences at *p* < 0.05 between the samples at different digestion phases (One-way Analysis of Variance followed by Duncan post hoc test). ^1^ total phenolic content (TPC); ^2^ Ascorbic acid equivalent antioxidant capacity (AAE), 1,1-diphenyl-2-picrylhydrazyl (DPPH); ^3^ 2,2′-Azino-bis (3-ethylbenzothiazoline-6-sulfonic acid) diammonium salt (ABTS). Y0: the 0% MCPP-fortified yogurt (as the control); Y0.1: 0.1% MCPP-fortified yogurt; Y0.5: 0.5% MCPP-fortified yogurt; Y1: 1% MCPP-fortified yogurt; Y2: 2% MCPP-fortified yogurt; M2: 2% MCPP water extract.

## Data Availability

The original contributions presented in this study are included in the article/Supplementary Materials. Further inquiries can be directed to the corresponding authors.

## Supplementary Materials

The following supporting information can be downloaded at: https://www.mdpi.com/article/10.3390/foods15081427/s1, Figure S1: Principal Component Analysis. (A) Three-dimensional distribution of samples based on the first three principal components. Each point represents a sample, with a color gradient indicating the value range of the target variable (component content %) from blue (low) to red (high). (B) PCA scree plot. Dual *y*-axis display of principal component variance contribution distribution. Left *y*-axis (blue bars): percentage of variance explained by each individual principal component, with specific values annotated above bars; Right *y*-axis (red line with circles): cumulative variance contribution rate. The green horizontal dashed line marks the 90% cumulative variance threshold; the green pentagram indicates the principal component position reaching this threshold (PCn, cumulative 94.8%); Table S1: Detailed model parameter configurations for machine learning; Table S2: Identification of volatile compounds by HS-GC-IMS of the stirred yogurt fortified with Mayang *Citrus sinensis* peel powder (MCPP); Table S3: Comprehensive importance ranking of key features; Table S4: Performance comparison of six machine learning models (100 bootstrap validations).
