# Supplementary material for: Targeted Prediction and Comprehensive Study of Stirred-Type Yogurt with Mayang Citrus Peel Powder Fortification Utilizing Machine Learning Approaches
Source: Foods. 2026 Apr 20;15(8):1427. doi: 10.3390/foods15081427 (PMC13116293; doi:10.3390/foods15081427)
Supplement: Supplementary file 1 [file foods-15-01427-s001.zip › Supplementary materials/Table S1.pdf]

Table S1 Detailed model parameter configurations for machine learning.

Model	Key Parameters	Detailed Configuration
SVM	Kernel=RBF, OptimizeHyperparameters=auto	KernelFunction: RBF, OptimizeHyperparameters: auto, AcquisitionFunction: expected-improvement-plus, ShowPlots: 0
XGBoost	NumTrees=100, MinLeafSize=1, Method=regression	NumTrees: 100, MinLeafSize: 1, Method: regression, NumPredictorsToSample: all
GBRT	NumLearningCycles=50, LearnRate=0.05, MinLeafSize=2, MaxNumSplits=10	NumLearningCycles: 50, LearnRate: 0.05, MinLeafSize: 2, MaxNumSplits: 10, Method: LSBoost
Ridge	Regularization=ridge, Lambda=auto, Solver=lbfgs	Regularization: ridge, Lambda: auto, Solver: lbfgs, Learner: leastsquares
Lasso	Regularization=lasso, CV=5fold, Lambda=minMSE	Regularization: lasso, CV: 5fold, Lambda: minMSE, Alpha: 1
ElasticNet	Regularization=ElasticNet, Alpha=0.5, CV=5fold, Lambda=minMSE	Regularization: ElasticNet, Alpha: 0.5, CV: 5fold, Lambda: minMSE
