# Supplementary material for: Targeted Prediction and Comprehensive Study of Stirred-Type Yogurt with Mayang Citrus Peel Powder Fortification Utilizing Machine Learning Approaches
Source: Foods. 2026 Apr 20;15(8):1427. doi: 10.3390/foods15081427 (PMC13116293; doi:10.3390/foods15081427)
Supplement: Supplementary file 1 [file foods-15-01427-s001.zip › Supplementary materials/Table S2.pdf]

Table S2 Identification of volatile compounds by HS-GC-IMS of **the** stirred yogurt fortified with Mayang *Citrus sinensis* peel powder (MCPP).

	Name of compounds	CAS#	RI[1]	Rt [sec][2]	Dt[3] [a.u.]	Peak intensity				
						Y0	Y0.1	Y0.5	Y1	Y2
Aldehydes	Hexanal	C66251	790.8	237.381	1.25967	194.00 ± 7.21^{e}	208.33 ± 5.03^{d}	269.67 ± 6.35^{c}	308.33 ± 7.23^{b}	378.33 ± 7.09^{a}
	2-Methylpropanal	C78842	526.7	124.317	1.26991	63.00 ± 6.56^{d}	142.67 ± 10.69^{c}	252.33 ± 10.97^{b}	282.67 ± 11.93^{a}	289.67 ± 3.21^{a}
	Propanal	C123386	491	116.401	1.17336	82.67 ± 4.04^{e}	836.67 ± 41.50^{d}	1590.00 ± 51.07^{c}	1887.33 ± 56.89^{b}	2203.33 ± 54.24^{a}
	Hex-2-enal	C505577	848.7	283.042	1.51749	65.67 ± 1.15^{d}	87.67 ± 1.15^{d}	470.00 ± 31.19^{c}	883.67 ± 8.39^{b}	1598.00 ± 8.89^{a}
Ketones	2-Butanone	C78933	591	141.855	1.24596	3992.00 ± 27.18^{b}	4057.67 ± 18.72^{a}	4058.33 ± 32.59^{a}	3960.00 ± 25.98^{b}	3873.00 ± 15.72^{c}
	2-Heptanone-D	C110430	889.6	322.333	1.63541	2218.67 ± 14.57^{a}	2238.67 ± 7.09^{a}	2176.33 ± 5.86^{b}	2167.67 ± 19.86^{b}	2159.33 ± 6.11^{b}
	2-Butanone 3-hydroxy	C513860	726.7	197.996	1.3291	2423.33 ± 82.40	2463.67 ± 26.39	2448.33 ± 15.28	2428.33 ± 34.15	2320.67 ± 35.81
	2,3-Pentanedione	C600146	608.2	147.395	1.29132	830.00 ± 70.70^{c}	898.33 ± 14.74^{ab}	962.67 ± 15.57^{a}	945.00 ± 18.33^{ab}	885.67 ± 8.02^{bc}
	2,3-Butandione	C431038	572.2	136.243	1.17417	1021.00 ± 35.54	1117.00 ± 151.83	1059.00 ± 118.88	1088.67 ± 169.00	1173.33 ± 94.69
	2-Pentanone-D	C107879	690.6	179.942	1.12531	824.33 ± 17.04^{a}	826.33 ± 19.86^{a}	810.67 ± 8.96^{a}	801.00 ± 16.70^{a}	768.67 ± 10.02^{b}
	2-Methyltetrahydrofuran-3-one	C3188009	785.1	233.456	1.41728	273.00 ± 14.00^{e}	303.00 ± 13.08^{d}	348.33 ± 14.15^{c}	373.00 ± 4.58^{b}	398.67 ± 5.69^{a}
	Mesityl oxide	C141797	797.3	242.036	1.44024	231.67 ± 3.21^{c}	235.67 ± 5.51^{c}	252.33 ± 2.31^{a}	245.00 ± 2.65^{b}	243.33 ± 3.21^{b}
	2-Hexanone-D	C591786	785.7	233.909	1.50262	428.00 ± 44.51^{a}	344.00 ± 8.00^{b}	292.00 ± 4.00^{c}	263.00 ± 6.00^{cd}	233.67 ± 3.21^{d}
	Methyl-5-hepten-2-one	C110930	985.6	445.049	1.18208	66.00 ± 1.73^{d}	68.67 ± 0.58^{d}	91.67 ± 1.53^{c}	146.67 ± 1.53^{b}	217.00 ± 5.20^{a}
Alcohols	Ethanol	C64175	455.2	109.528	1.11963	3226.67 ± 24.91^{b}	3343.67 ± 68.24^{a}	3353.67 ± 17.24^{a}	3290.33 ± 29.37^{ab}	3246.00 ± 26.89^{b}
	1-Butanol 2-methyl	C137326	720.8	194.865	1.23668	607.00 ± 39.51^{a}	621.67 ± 9.50^{a}	608.33 ± 12.01^{a}	592.67 ± 13.20^{a}	542.67 ± 11.59^{b}
	(Z)-2-Pentenol	C1576950	783.9	232.642	1.44721	234.67 ± 26.01^{ab}	208.00 ± 4.36^{c}	209.33 ± 2.52^{c}	224.00 ± 4.58^{bc}	255.00 ± 4.36^{a}
	1-Propanethiol	C107039	622.2	152.159	1.18064	403.00 ± 22.72^{a}	307.67 ± 51.63^{b}	235.00 ± 8.72^{c}	229.67 ± 8.39^{c}	252.67 ± 9.61^{c}
	1-Butanol	C71363	666.6	169.313	1.18232	232.00 ± 17.58^{e}	259.00 ± 3.61^{d}	435.33 ± 10.07^{c}	659.67 ± 3.21^{b}	898.67 ± 12.42^{a}
	n-Hexanol	C111273	871.4	304.072	1.63996	70.33 ± 1.53^{d}	73.00 ± 3.00^{d}	102.00 ± 3.46^{c}	178.33 ± 4.62^{b}	335.00 ± 11.00^{a}
Acids	Butanoic acid	C107926	800.4	244.245	1.38309	225.67 ± 24.83^{b}	261.00 ± 5.00^{a}	255.67 ± 7.77^{a}	250.67 ± 5.69^{a}	243.33 ± 6.66^{ab}
	Pentanoic acid	C109524	920.4	356.626	1.22096	74.33 ± 0.58^{d}	76.00 ± 2.00^{d}	101.00 ± 2.65^{c}	149.67 ± 4.73^{b}	205.67 ± 6.43^{a}
Esters	Ethyl heptanoate	C106309	1097.2	662.884	1.41202	174.00 ± 30.35	154.67 ± 3.51	150.00 ± 2.00	153.00 ± 3.61	148.67 ± 2.08
	Isopropyl acetate	C108214	658.5	165.931	1.15852	224.00 ± 6.00^{d}	236.00 ± 11.27^{d}	316.00 ± 2.65^{c}	359.67 ± 7.37^{b}	420.67 ± 8.14^{a}
	Methyl hexanoate	C106707	925.3	362.465	1.29223	65.00 ± 2.00^{d}	68.00 ± 1.00^{d}	103.00 ± 2.65^{c}	172.67 ± 3.21^{b}	287.33 ± 4.16^{a}
	Sotolone	C28664359	1101.6	673.614	1.22528	55.00 ± 2.65^{d}	55.67 ± 2.89^{d}	70.00 ± 2.00^{c}	99.67 ± 4.51^{b}	153.00 ± 3.00^{a}
alkenes	alpha-terpinene	C99865	1015.7	494.479	1.22172	56.67 ± 2.52^{d}	253.00 ± 4.58^{c}	743.33 ± 10.50^{b}	860.67 ± 4.62^{a}	861.67 ± 3.06^{a}
	beta-Myrcene-M	C123353	984.2	442.924	1.2186	73.33 ± 1.53^{d}	76.33 ± 1.53^{d}	141.33 ± 4.51^{c}	326.67 ± 4.04^{b}	503.00 ± 2.65^{a}
	Diethylene glycol dimethyl ether	C111966	949.6	393.33	1.15616	167.67 ± 9.07^{bc}	161.33 ± 8.62^{bc}	157.67 ± 6.11^{c}	170.67 ± 4.16^{b}	199.33 ± 3.06^{a}

Others	p-Cymene	C99876	1017.3	497.127	1.30161	63.00 ± 1.00[e]	250.00 ± 12.00[d]	979.00 ± 22.61[c]	1628.33 ± 5.51[b]	1839.67 ± 9.61[a]
	2-Ethyl-5-methylpyrazine	C13360640	1014.9	493.007	1.65878	87.00 ± 2.65[d]	87.67 ± 2.31[d]	153.00 ± 6.08[c]	388.67 ± 4.62[b]	556.67 ± 4.93[a]
	1,4-Cineole	C470677	1017.1	496.768	1.71123	81.67 ± 1.15[d]	80.00 ± 0.00[d]	107.33 ± 1.53[c]	203.00 ± 5.00[b]	274.67 ± 2.52[a]

All values are means ± SD (n = 3). Different lowercase letter superscripts within the same line indicate statistically significant differences at $p < 0.05$ between the yogurts fortified with different amounts of MCPP (One-way Analysis of Variance followed by Duncan post hoc test). [1] RI: the retention index (experimental value). [2] Rt [sec]: the retention time. [3] Dt [a.u.]: the drift time. Y0: the 0% MCPP-fortified yogurt (as the control); Y0.1: 0.1% MCPP-fortified yogurt; Y0.5: 0.5% MCPP-fortified yogurt; Y1: 1% MCPP-fortified yogurt; Y2: 2% MCPP-fortified yogurt.
