# Supplementary material for: Targeted Prediction and Comprehensive Study of Stirred-Type Yogurt with Mayang Citrus Peel Powder Fortification Utilizing Machine Learning Approaches
Source: Foods. 2026 Apr 20;15(8):1427. doi: 10.3390/foods15081427 (PMC13116293; doi:10.3390/foods15081427)
Supplement: Supplementary file 1 [file foods-15-01427-s001.zip › Supplementary materials/Table S3.pdf]

Table S3 Comprehensive importance ranking of key features.

Principal Component	Top1_Feature_Loading	Top2_Feature_Loading	Top3_Feature_Loading	Top4_Feature_Loading	Top5_Feature_Loading	Variance Explained
PC1 (79.9%)	TPC (0.275)	AC_{ABTS} (0.273)	L* (-0.271)	b* (0.270)	TA (0.264)	79.89%
PC2 (9.7%)	AC_{DPPH} (0.430)	a* (0.351)	pH (0.349)	LAB cells (-0.328)	Firmness (0.324)	9.73%
PC3 (5.2%)	Viscosity index (0.573)	Viscosity (0.533)	Syneresis (0.319)	WHC (-0.319)	Cohesiveness (0.270)	5.18%
PC4 (2.5%)	pH (0.728)	WHC (-0.408)	Syneresis (0.408)	Consistency (-0.273)	L* (0.157)	2.48%
PC5 (1.2%)	TA (0.570)	Consistency (-0.341)	Viscosity (0.288)	Firmness (-0.285)	Viscosity index (-0.280)	1.16%
