# Supplementary material for: Targeted Prediction and Comprehensive Study of Stirred-Type Yogurt with Mayang Citrus Peel Powder Fortification Utilizing Machine Learning Approaches
Source: Foods. 2026 Apr 20;15(8):1427. doi: 10.3390/foods15081427 (PMC13116293; doi:10.3390/foods15081427)
Supplement: Supplementary file 1 [file foods-15-01427-s001.zip › Supplementary materials/Table S4.pdf]

Table S4 Performance comparison of six machine learning models (100 bootstrap validations).

Model	R^2	RMSE	MSE	Rank	Combined Score
SVM	0.4575	0.0028	1.691×10^{-5}	6	0
XGBoost	0.7848	0.0024	9.542×10^{-6}	5	0.4571
GBRT	0.8175	0.0015	5.124×10^{-6}	4	0.6924
Ridge	0.9217	0.0013	2.155×10^{-6}	3	0.9174
Lasso	0.9265	0.0011	1.395×10^{-6}	1	0.9975
ElasticNet	0.9284	0.0012	1.927×10^{-6}	2	0.9460
