# Supplementary figures and images for: Targeted Prediction and Comprehensive Study of Stirred-Type Yogurt with Mayang Citrus Peel Powder Fortification Utilizing Machine Learning Approaches
Source: Foods. 2026 Apr 20;15(8):1427. doi: 10.3390/foods15081427 (PMC13116293; doi:10.3390/foods15081427)

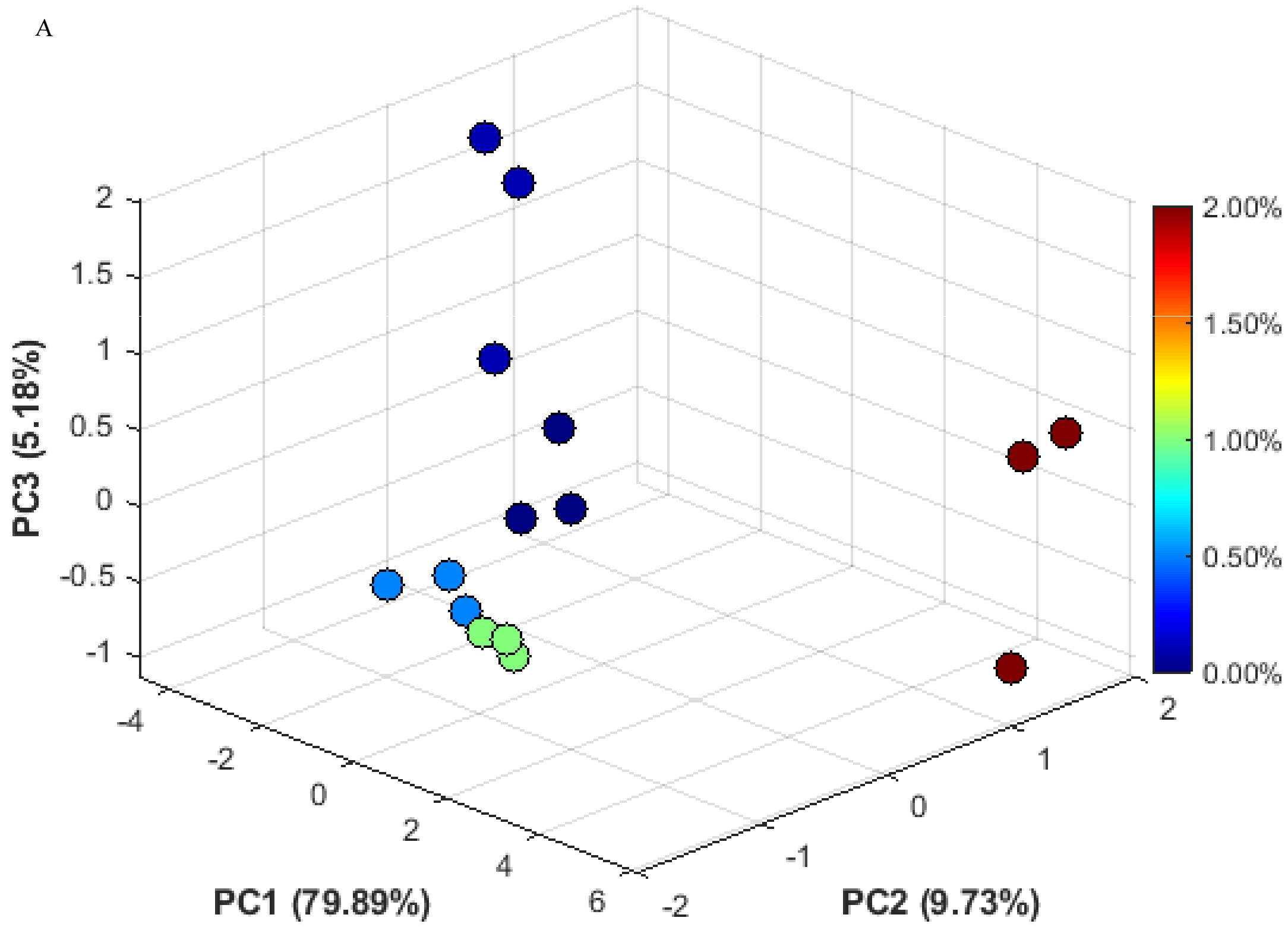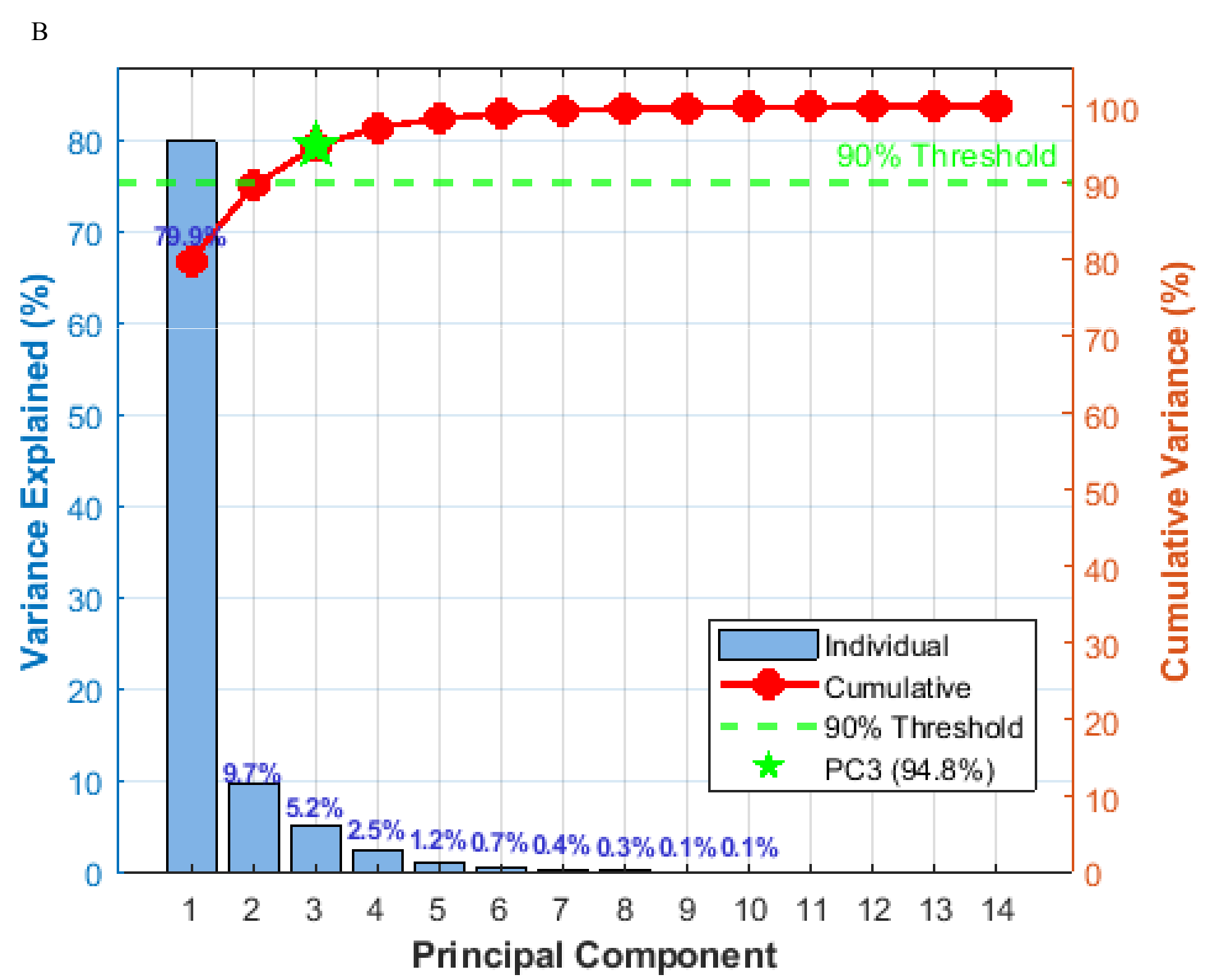

Supplement: Supplementary file 1 [file foods-15-01427-s001.zip › Supplementary materials/Figure S1.pdf]
